# Analysis of emotional prosody as a tool for differential diagnosis of cognitive impairments: a pilot research

**DOI:** 10.3389/fpsyg.2023.1129406

**Published:** 2023-06-22

**Authors:** Chorong Oh, Richard Morris, Xianhui Wang, Morgan S. Raskin

**Affiliations:** ^1^School of Rehabilitation and Communication Sciences, Ohio University, Athens, OH, United States; ^2^School of Communication Science and Disorders, Florida State University, Tallahassee, FL, United States; ^3^School of Medicine, University of California Irvine, Irvine, CA, United States

**Keywords:** dementia, mild cognitive impairment, emotion, prosody, acoustic analysis, listener perception, diagnosis

## Abstract

**Introduction:**

This pilot research was designed to investigate if prosodic features from running spontaneous speech could differentiate dementia of the Alzheimer’s type (DAT), vascular dementia (VaD), mild cognitive impairment (MCI), and healthy cognition. The study included acoustic measurements of prosodic features (Study 1) and listeners’ perception of emotional prosody differences (Study 2).

**Methods:**

For Study 1, prerecorded speech samples describing the *Cookie Theft* picture from 10 individuals with DAT, 5 with VaD, 9 with MCI, and 10 neurologically healthy controls (NHC) were obtained from the DementiaBank. The descriptive narratives by each participant were separated into utterances. These utterances were measured on 22 acoustic features *via* the Praat software and analyzed statistically using the principal component analysis (PCA), regression, and Mahalanobis distance measures.

**Results:**

The analyses on acoustic data revealed a set of five factors and four salient features (i.e., pitch, amplitude, rate, and syllable) that discriminate the four groups. For Study 2, a group of 28 listeners served as judges of emotions expressed by the speakers. After a set of training and practice sessions, they were instructed to indicate the emotions they heard. Regression measures were used to analyze the perceptual data. The perceptual data indicated that the factor underlying pitch measures had the greatest strength for the listeners to separate the groups.

**Discussion:**

The present pilot work showed that using acoustic measures of prosodic features may be a functional method for differentiating among DAT, VaD, MCI, and NHC. Future studies with data collected under a controlled environment using better stimuli are warranted.

## Introduction

Currently, diagnosis of cognitive impairments relies heavily on invasive (e.g., lumbar puncture) and/or expensive (e.g., neuroimaging panel) biomarker tests (López-de-Ipiña et al., 2015). The results of biomarker tests, primarily obtained using invasive lumbar punctures, depend significantly on the patient’s physical health and age, which decreases the efficacy of the method (Maclin et al., 2019). Expensive neuroimaging lacks definitive characteristics with significant diagnostic value (Filippi et al., 2012) which decreases the diagnostic accuracy, and many patients experience claustrophobia, discomfort, or behavioral problems during the imaging sessions and cannot tolerate them (Bonifacio and Zamboni, 2016). These issues lead to the decreased diagnostic accuracy and eventually the overall costs for dementia care increase not only because of the high cost and invasive nature of the exams but also because of the extensive clinical testing that often takes place while individuals seek opinions from multiple providers regarding the source of their symptoms before ultimately reaching a provider in a facility that has access to these diagnostic exams. The extended time increases both personal and monetary costs associated with dementia diagnosis, which subsequently increases financial burden on people with cognitive impairment, families, and society and also delays the initiation of proper care.

Speech and language production requires coordination among highly complicated and calibrated brain systems, including but not limited to Broca’s and Wernicke’s areas. When the coordination is not accomplished properly due to a brain disease or accident, it may yield significant changes in the person’s speech and/or language functions. People with cognitive impairment such as dementia demonstrate various speech and language deficits. While linguistic deficits such as word finding difficulty and agrammatism are well documented and have been used to identify early-stage cognitive declines (e.g., Lundholm Fors et al., 2018; Calzá et al., 2021), data on speech deficits in people with different types of cognitive impairment are limited. It should also be noted that speech and language deficits are not clearly distinguished in the dementia literature; often, language deficits are misinterpreted as speech deficits or the two terms (i.e., speech impairment and language impairment) are used interchangeably. However, the distinction between speech and language impairments is critical to understanding any impaired communication functioning and for making more accurate diagnoses and creating appropriate management plans.

The use of vocal biomarker may provide useful information for diagnosis and monitoring of different diseases/disorders as well as for phenotyping a condition (Fagherazzi et al., 2021). Among many voice features, prosody is an aspect of speech that consists of perceptible suprasegmental modulations of vocal pitch, syllable length, loudness, and pauses (Odell et al., 1991). These modulations deliver the speaker’s meaning beyond the literal meaning of the utterance and give the listener clues to interpret the connotative meaning intended by the speaker (Hupp and Junger, 2013). The manipulation of prosody requires a wide range of interhemispheric cerebral networks, which are impaired in people with cognitive impairment to different extents depending on the type of condition (Lian et al., 2018; Qi et al., 2019; Cheung and Mak, 2020). For instance, the accumulation of amyloid fibrils decreases interhemispheric functional connectivity (IFC) in visual network for dementia of the Alzheimer’s type (DAT) while it increases with the IFC in default mode network, central executive network, sensory motor network, and dorsal attention network for vascular dementia (VaD) (Cheung and Mak, 2020). Such differences suggest that prosody, of which manipulation is completed *via* interhemispheric connectivity, may be an effective, reliable, and low-cost method to differentiate cognitive impairment types. In particular, the emotional aspects of prosody (i.e., expression of emotion through variations of different parameters of speech) provide a method for the speaker to utter a nuanced message that can be accurately perceived by a listener and may vary systematically with the expression of emotion (Pell et al., 2009). However, the available data on emotion expression in people with different types of cognitive impairment and neurotypical speakers are sparse.

The production of the prosodic features involves movement variations in all components of the speech production mechanism (Pell et al., 2009). Thus, changes in these acoustic measures may represent changes in the motor system associated with the neurologic changes associated with the different dementia types. In a review of cognitive, psychiatric, and motor symptoms of different dementia types, Magdy and Hussein (2022) reported that motor symptoms were significant indicators for Parkinson disease related dementias (e.g., corticobasal degeneration, dementia with Lewy bodies, and multiple system atrophy), normal pressure hydrocephalus, frontotemporal dementias and the posterior cortical atrophy variant of DAT. People with mild cognitive impairment (MCI) and DAT exhibit motor issues for complex tasks that can distinguish them from neurologically healthy controls (NHC) (Kluger et al., 1997). Although early-stage VaD and MCI can have similar cognitive symptoms, people with early-stage VaD do not tend to have motor symptoms (Kandasamy et al., 2020). The specific patterns of the motor issues relative to speech production for people with MCI and DAT have not been specifically described. Quite possibly, these motor issues may differ among the dementia types. Thus, the prosodic patterns for expressing emotion may provide a means to explore differences among cognitive impairment types.

Acoustic measurements that comprise prosody, such as fundamental frequency (f0), amplitude measured in dB level, and speech rate have been associated with the vocal expressions of emotions (Scherer, 2003), and several authors have reported evidence for emotion-specific patterns of acoustic cues (Banse and Scherer, 1996; Juslin and Laukka, 2003; Hamnmerschmidt and Jurgens, 2007). Mean f0 tends to be high (with a fast speech rate) for happiness, fear, and anger, and low for sadness (with a slow speech rate). F0 variability tends to be wide for happiness and anger but narrow for fear and sadness (Juslin and Laukka, 2003). Listeners exhibit approximately 60% accuracy for recognizing emotion from voice samples, although some emotions with more distinctive acoustic profiles (such as sadness and anger) may be easier for raters to identify than others (Johnstone and Scherer, 2000). However, this issue is complicated as the acoustic features of “emotional” prosody are not clear, given that there is no consensus on how acoustic features are manipulated to express different “emotions” (c.f., Bulut and Narayanan, 2008). For example, it is unclear how the frequency, amplitude, duration, and/or spectrum measures change when a person is in a state of emotional arousal, compared to when s/he is not (Patel et al., 2011). Without this discussion, the investigations into emotional prosody cannot be complete.

The present investigation, thus, was designed to provide preliminary evidence of unique prosodic production profiles of people with three types of cognitive impairment: DAT, VaD, and MCI (Study 1). Specifically, it was aimed to clarify how prosodic features differ acoustically across people with DAT, VaD, MCI, and healthy cognition and to determine whether the patterns of prosodic features can be used to differentially diagnose DAT, VaD, and MCI. One important concern of this study was whether these prosodic features could be associated with the expression of emotion. Accordingly, the categorization of perceived acoustic features into emotional versus non-emotional, or neutral, prosody was also carried out (Study 2). It was hypothesized that (1) the types of cognitive impairments will be associated with different prosodic features in comparison to neurotypical older adults and (2) unique patterns of emotion expression will be perceived for each group by neurotypical listeners. Overall, it was expected that the different prosodic features could lead to a useful tool for differential diagnosis of DAT, VaD, and MCI.

## Methods

### Procedures

#### Study 1 – Acoustic analysis of emotional prosody

##### Materials

For the first purpose, audio recordings of people with DAT, VaD, MCI, and NHC were obtained through DementiaBank,^1^ a shared database supported by NIH-NIDCD grant R01-DC008524. The use of the secondary data was approved by Institutional Review Board at Ohio University (21-X-74). Included in this dataset were 10 people with DAT, 9 with MCI, 5 with VaD, and 10 NHC. On average, the speakers were 66.4 years old with 13.97 years of education at the time of original data collection. The one-way analysis of variance (ANOVA) revealed that across the four groups, the level of education (*F*(3, 31) = 1.791, *p* = 0.169) and age (*F*(3, 31) = 2.094, *p* = 0.121) of participants were not significantly different but the difference in Mini Mental State Exam (MMSE) scores were significant (*F*(3, 31) = 34.761, *p* < 0.001). Detailed characteristics of the speakers can be found in Table 1. Among the speech samples available to DementiaBank members, those describing the *Cookie Theft* picture in English from the *Pitt* (Becker et al., 1994) corpus were used based on previous research showing that the *Cookie Theft* picture description task, from the Boston Diagnostic Aphasia Examination (Goodglass et al., 2001), provides a rich context in which mental state language and the cognitive processes associated with this language can be investigated (Cummings, 2019). It has been used to determine atypical emotional prosodic features of different clinical populations: Villain et al. (2016) found that stroke survivors described the picture using atypical emotional prosodic patterns, which is indicative of post-stroke depression. Wright et al. (2018) also reported atypical emotional prosody when describing the picture in right hemisphere stroke survivors and Patel et al. (2018) provided MRI images supporting the atypical prosodic patterns in this population. In individuals with dementia, Nevler et al. (2017) found that the Cookie Theft picture description task evoked emotional responses in people with behavioral variant frontotemporal dementia. Similarly, Haider et al. (2020) demonstrated that when using the *Cookie Theft* picture description task with a focus on emotional prosody, the accuracy of detecting Alzheimer’s disease was 63.42%, which is comparable to when using the Berlin Database of Emotional Speech.

**Table 1:** Speaker demographics.

Group	Mean years of age^a^ (σ)	Sex (men, women)	Mean years of education (σ)	Mean MMSE^b^ score (σ)
NHC	63.00 (9.24)	2, 8	14.9 (2.56)	29.3 (1.16)
DAT	69.36 (5.90)	4, 6	13.45 (3.47)	17.91 (5.54)
VaD	72.6 (6.12)	2, 3	11.2 (2.71)	15.4 (1.74)
MCI	63.11 (11.22)	5, 5	14.78 (3.42)	27.89 (1.45)

a Standard deviation.

b Mini mental state exam (score range: 0–30).

##### Acoustic analysis

The audio recordings and accompanying transcripts were downloaded and saved. The transcripts were compared to the audio files and amended as needed. Most amendments consisted of adding repetitions and filled pauses. The audio files were then parsed into utterances by the first and second authors of the current research independently, considering pauses and connectivity. After the independent work, the two researchers compared their evaluations and disagreements were resolved *via* discussions, until they reached 100% agreement. This parsing process resulted in a final outcome of 365 utterances including 108 utterances in the DAT, 75 in the MCI, 49 in the VaD, and 133 in the NHC groups. The utterances were then analyzed acoustically using the Praat software (Boersma and Weenink, 2017, v. 6.1.14) via a set of timing, pitch, and amplitude measures.

For timing, the following set of measurements was made for each utterance: the duration of the complete utterance including pauses and repetitions. This measure was recorded as the speech time. Then, the pauses longer than 200 ms and filled pauses, word repetitions, and syllable repetitions were removed from the utterances and the duration of the remaining signal was measured. This measure was recorded as the articulation time. In addition, the number of syllables in the utterance and the number of repeated syllables and repeated words were recorded. Finally, the duration of the removed pauses and duration of the repeated syllables and words were recorded. The speech time was divided by the total number of syllables, repeated syllables, and repeated words to determine the speech rate in syllables per second. The articulation time was divided by the number of syllables in the utterance to determine the articulation rate.

Many of these duration, timing, and extra syllable measures have indicated differences in expressed emotions. Comparisons between neutral and emotional speech have revealed that syllable and word repetitions decrease for emotional speech (Buchanan et al., 2014). Tao et al. (2018) reported that nonlinguistic fillers have no lexical information but contain emotional information. In addition, sad and fearful emotions are produced with more pauses, in comparison to neutral speech (Sauter et al., 2010). When rates have been calculated, they carry emotional valence as speaking rate differs among happiness, anger, sadness, fear, and neutral and articulation rate is slower for negative emotions (Petrushin, 1999; Erdemir et al., 2018; Tao et al., 2018).

After completing the utterance rate measures, the waveform of the articulation time for each utterance was displayed and the voiceless segments were removed using hand-controlled cursors to mark the voiceless segments. This version of the utterance was used for the pitch, loudness, and LTAS measures.

For pitch, the following set of f0 measurements were made for each utterance: the f0 of the first stable cycle of the first voiced sound and the f0 of the last stable cycle of the final voiced sound. In addition, the following measurements were collected using the output from the Voice Report from the Pulse menu in Praat: the highest f0 in the utterance, the lowest f0 in the utterance, and the median f0. The median f0 was used to reduce the effects of possible wide upward f0 shifts on the mean f0. The minimum f0 was subtracted from the maximum f0 to determine the range of f0 used (Δf0).

Similar to the duration and timing measures, the frequency measures have been used to differentiate among emotions and f0 measures considered to be primary indicators of emotional prosody (Bulut and Narayanan, 2008; Patel et al., 2011). Fear, joy, and anger are portrayed at a higher f0 than sadness and the f0 extent differs between happiness and fear (Bachorowski, 1999; Paeschke and Sendlmeier, 2000). The initial f0 differs between anger and sadness and the final f0 differs between happiness and sadness (Paeschke and Sendlmeier, 2000; Sauter et al., 2010). Finally, the average f0 differs between happiness and sadness (Paeschke and Sendlmeier, 2000).

For the loudness of the speech in dB (SPL), the following set of measurements was made for each utterance: the SPL of the first stable cycle of the first voiced sound and the SPL of the last stable cycle of the final voiced sound. In addition, the following measurements were collected using the output from the Intensity menu in Praat: the highest SPL in the utterance, the lowest SPL in the utterance, and the average SPL.

In comparison to the previous two sets of measures, measures of the relationship between SPL and emotion have been less explored. The average SPL differs between fear and sadness (Tao et al., 2006). In addition, the extent of SPL variations differs between anger and happiness (Tao et al., 2006). Since the SPL extent is determined from the maximum and minimum SPL levels, these measures may individually mark emotional differences. Similarly, the initial and final SPL levels may mark emotional differences.

Finally, three long-term average spectral (LTAS) measurements were made using the utterances without the voiceless segments: the LTAS slope, the LTAS offset, and the LTAS alpha ratio. These were extracted using standard bandwidth settings in the Praat LTAS routines. The LTAS measures indicate the pattern of amplitude by frequency. This interaction has indicated differences in emotional prosody as LTAS differences have been reported between sadness and anger and these measures mark the strength of emotional prosodic change (Tao et al., 2006; Cole et al., 2007).

#### Study 2 – Listener perception of emotional prosody

Study 2 was aimed at providing data to define “emotional” prosody to be used for differential diagnosis of cognitive impairments: when do listeners perceive emotion and what acoustic features are associated with the specific emotion? Neurotypical native English users were recruited to evaluate emotions expressed in each of the utterances per the approval of Institutional Review Board at Ohio University (21-X-61). The listeners were tested for their cognitive functioning using the Montreal Cognitive Assessment (MoCA; Nasreddine et al., 2005) and only those who scored above 26 (out of 30) were allowed to participate in the emotion evaluation.

For the emotion evaluation, a perception experiment consisting of practice, screening, and main sessions, was built online with Gorilla™.^2^ The practice session was offered to anchor the listeners’ evaluation using pseudo examples of seven emotions (i.e., happiness, sadness, disappointment, fear, surprise, anger, and neutral), developed and validated by Pell et al. (2009). During the practice trials, each listener was asked to choose the emotion of each utterance spoken by a professional actor or actress from seven choices including the 6 emotions mentioned above and a “neutral” option. The practice session consisted of 70 trials (with each of the six emotions and “neutral” appearing 10 times in random order) and feedback was provided following each response. After the practice session, each listener was asked whether s/he was confident to proceed to the screening test, which was shorter (10 trials) but followed the same format as the practice session. If the listener was not self-assured, another round of practice using a different set of utterances would be offered. A participant was considered passing the screening when s/he correctly identified at least 7 out of the 10 utterances. Failing the screening test would lead to an extra session of practice followed by a second screening test with a different set of utterances. Those who made two successive failures in the screening test would be excluded from participation. A total of 51 listeners participated in the screening test: 13 of them did not complete the screening and 28 of the 38 who completed the screening passed the screening at the pass rate of 73.6%. On average, the listeners were 29.6 years old (σ = 11.62) with 15.67 years of education (σ = 1.75) and earned 27.9 (σ = 1.30) on MoCA. Fourteen of them were men.

These 28 listeners, who successfully passed the screening, then moved on to the main test, where they were instructed to judge the emotions expressed in the *Cooke Theft* description utterances obtained from the DementiaBank. The listeners were informed that no feedback would be provided during the test. They were also instructed to make their best judgments based on their knowledge gained through the practice and screening sessions.

### Statistical analysis

All statistical analyses were done using R version 4.1.0. The acoustic measures in Study 1 were analyzed using a principal component analysis (PCA) to determine the separate factors and grouping of the acoustic measures and a regression model to determine the acoustic measures representing a unique aspect of the variance across the cognitive impairment types. The criteria used as the probability to for entering additional terms to the model was set at less than or equal to *p* = 0.05. Finally, a Mahalanobis distance measure for multivariate ANOVA to determine how well the factors discriminated among the cognitive impairment types.

The utterances used in acoustic measures were then categorized into different emotions based on the perceptual evaluations by listeners in Study 2. Specifically, the counts for all emotions were obtained for each utterance, and that utterance was labelled as the emotion with the most counts. For example, if an utterance was perceived as “Angry” by 10 listeners and “Sad” by 3 listeners, that utterance would be labelled as “Angry.” Utterances classified to the same emotion were then calculated for the descriptive statistics (i.e., mean and standard deviation) for each acoustic measure. A logistic regression model was constructed with the factor scores for each factor identified in the PCA for all utterances as independent variables and the emotion as the dependent variable. The emotion was coded into two classes: either neutral or emotional. The neutral class included utterances that were perceived as ‘Neutral’, and the emotional class included those identified as the rest 6 types of emotions.

## Results

### Study 1 – Acoustic analysis of emotional prosody

#### Factor analysis

**Figure 1:**
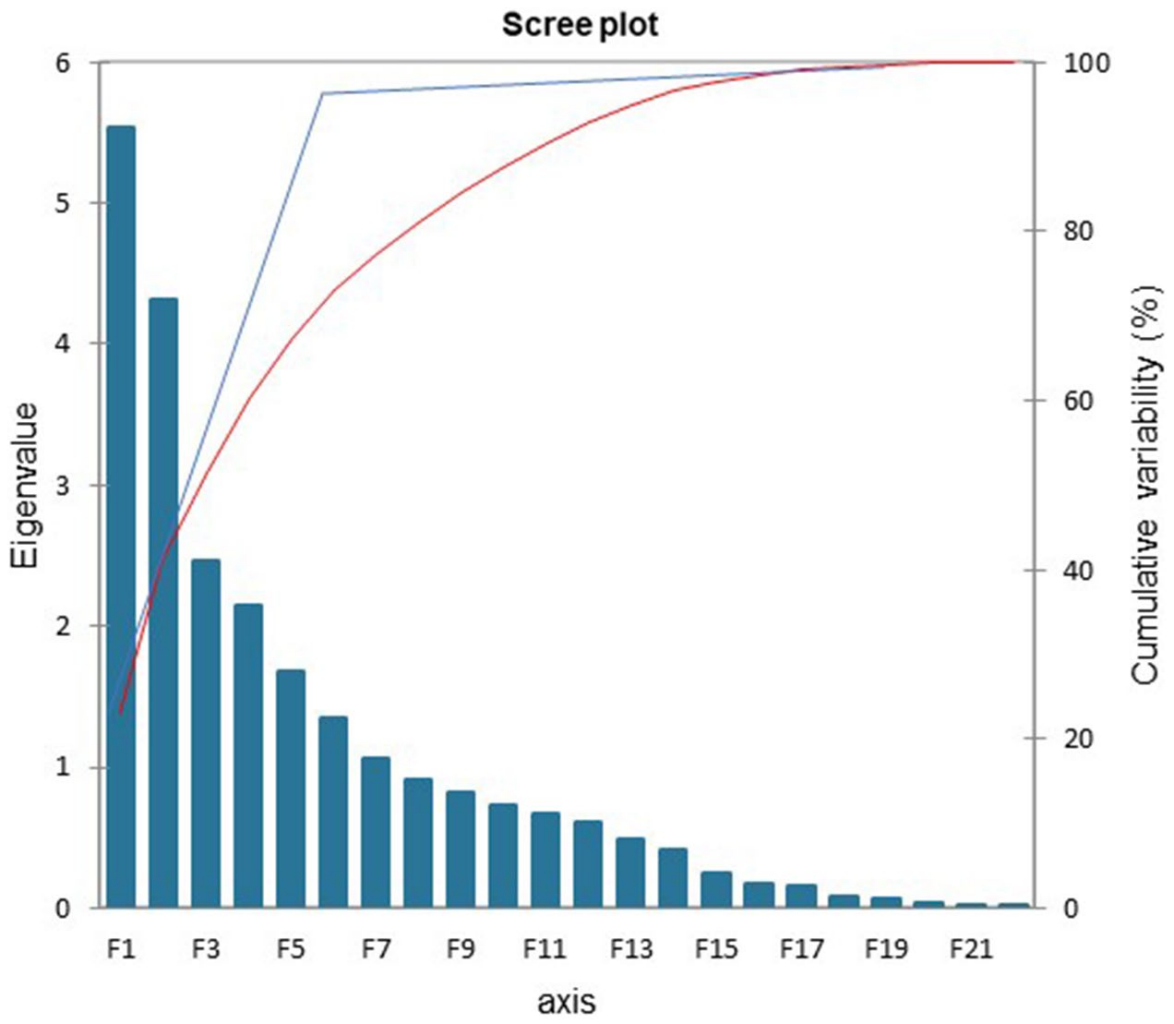
Scree plot: factors accounted for 67% of the overall variance.

The PCA was used to identify a small number of factors to represent relationships among sets of interrelated variables. The factor analysis of the acoustic measures revealed five factors with eigenvalues greater than 1.5. These factors and the included acoustic measures are depicted in Figure 1. The eigenvalues indicated the total variance explained by the correlated acoustic measures that comprise each factor. Two aspects of the data supported this stopping point for factors to include: first the variability accounted for dropped from 7 to 5.5% and second, the cumulative variability flattened after factor 5, as displayed in the scree plot in Figure 1. The five-factor model explained 67% of the total variance among the acoustic measures when separating the cognitive impairment types.

Acoustic measures were considered components of a factor when the factor loading was greater than 0.5 (Table 2). The first factor, labeled ‘Mixed’, was comprised of the following acoustic measures: the number of syllables for the speech and the articulation measures, the change in dB level, and the elapsed time for the speech and the articulation samples. The second factor was labeled ‘Loudness’ and included the initial, final, maximum, and minimum dB levels. The third factor was labeled ‘Pitch’ and was comprised of the final and maximum fundamental frequency levels as well as the difference in fundamental frequency level within each sample. The fourth factor is titled ‘Rate’ and included the speech and articulation rates. The fifth factor included the extra syllable count and extra syllable time and was labeled ‘Syllable.’ The extra syllables were repetitions and filled pauses. The difference between the acoustic measures in Factor 1 and Factor 4 was as follows: In Factor 1 the measures for speech and articulation are the number of syllables in the utterances and the elapsed time for each of those. In Factor 4, the acoustic measures are the division of the number of syllables by the elapsed time. It is noteworthy that these arithmetically related acoustic measures represented different aspects and proportions of the total variance of the differences among the four cognitive impairment groups.

**Table 2:** Output of the principal component factor analysis including the correlation between the acoustic measures and the factors, the factor eigenvalues, and the percentage of variance explained by each factor.

Construct	Loadings	Factors
		1	2	3	4	5
Mixed	Speech syllables	0.818				
	Speech time	0.837				
	Articulation syllables	0.802				
	Articulation time	0.849				
Loudness	dB change	**0.732**				
	dB initial		0.780			
	dB final		0.806			
	dB maximum		0.715			
	dB minimum		0.883			
Pitch	Frequency initial			0.559		
	Frequency final			**0.645**		
	Frequency maximum			0.792		
	Frequency change			0.708		
Rate	Speech rate				0.733	
	Articulation rate				**0.805**	
Syllable	Extra syllables					0.702
	Sum of extra syllables					**0.704**
	Eigenvalues	5.542	4.322	2.469	2.145	1.686
	Variance percentage	23.093	18.009	10.289	8.936	7.024

The acoustic measures included in the model created by the stepwise regression are in bold type.

#### Regression model

The stepwise fixed effects regression resulted in the inclusion of the following acoustic measures: the fundamental frequency at the end of the utterances, the articulation rates, the change in dB level during the utterance, and the sum of extra syllables in the utterances (Table 3). These acoustic measures were loaded onto separate factors in the factor analysis. The fixed effects regression model summary showed that all four measures were attributed to a significant amount of the total variance of the acoustic measures in relation to the cognitive impairment types. The negative Beta values for change in dB level within the utterance and articulation rate indicate that reductions in these two acoustic measures differentiated the cognitive impairment types. Finally, the tolerance information in the fourth model indicates that the variance explained by each of the acoustic measures was independent of the variance explained by the other acoustic measures included in the model.

**Table 3:** Results of stepwise regression including the four models, the *R*^2^ explained, and the *R*^2^ change for each model.

Variable	Beta (standard error)	*t*	*p*	95% CI [lower]^a^	95% CI [upper]	Tolerance
Model 1 (*R*^2^ = 0.081, *R*^2^ change = 0.081)						
(Constant)		12.336	<0.001	1.287	1.776	
Frequency final	0.284 (0.001)	5.635	<0.001	0.003	0.005	1.000
Model 2 (*R*^2^ = 0.1451, *R*^2^ change = 0.064)						
(Constant)		11.365	<0.001	2.052	2.91	
Frequency final	0.277 (0.001)	5.691	<0.001	0.003	0.005	0.999
Articulation rate	−0.254 (0.041)	−5.204	<0.001	−0.294	−0.133	0.999
Model 3 (*R*^2^ = 0.163, *R*^2^ change = 0.017)						
(Constant)		10.51	<0.001	2.425	3.541	
Frequency final	0.279 (0.001)	5.772	<0.001	0.003	0.005	0.999
Articulation rate	−0.281 (0.042)	−5.694	<0.001	−0.318	−0.155	0.959
dB change	−0.135 (0.007)	−2.733	0.007	−0.034	−0.006	0.959
Model 4 (*R*^2^ = 0.174, *R*^2^ change = 0.012)						
(Constant)		10.771	<0.001	2.504	3.622	
Frequency final	0.284 (0.001)	5.898	<0.001	0.003	0.005	0.997
Articulation rate	−0.296 (0.042)	−5.981	<0.001	−0.331	−0.167	0.941
dB change	−0.164 (0.008)	−3.232	0.001	−0.039	−0.010	0.898
Sum of extra syllables	0.113 (0.139)	2.271	0.024	0.042	0.587	0.928

a Confidence interval.

#### Multivariate distance model

A set of Mahalanobis distance tests were completed. The Mahalanobis distance shows how far the test point is from the benchmark point. A Malahanobis distance of 1 or lower indicates that the test point is similar to the benchmark point. These measures indicate the distance between selected points in multivariate space. The Mahalanobis distance tests revealed that all of the factors exhibited relatively weak sensitivity; however, they exhibited good specificity. Although the discriminatory sensitivity was weak, the Mahalanobis distance factors had separate patterns across the cognitive impairment groups. The first, ‘Mixed,’ factor separated the DAT group from the other three factors (*F*(21,1,020) = 4.259, *p* < 0.001) with Mahalanobis distances that ranged from 0.423 to 1.06 which included the sum of extra syllables from the regression analysis. The second, ‘Loudness,’ factor distinguished the participants in the NHC and VaD groups from those in the DAT and MCI groups (*F*(15,986) = 3.489, *p* < 0.001) with Mahalanobis distances ranging from 0.231 to 0.773. ‘Loudness’ included the change in dB level during the utterance acoustic measurement from the regression analysis. The third, ‘Pitch,’ factor then separated the DAT and MCI groups from the NHC and VaD groups (*F*(9,874) = 9.378, *p* < 0.001) with Mahalanobis distances that ranged from 0.119 to 1.603 which included the final fundamental frequency measure from the regression analysis. The fourth, ‘Rate,’ factor included the articulation rate measure from the regression analysis which separated the DAT and NHC groups from the MCI and VaD groups (*F*(6,720) = 4.579, *p* < 0.001) with Mahalanobis distances of 0.008 to 0.438. The final, ‘Syllable,’ factor separated the NHC group from the three cognitive impairment groups (*F*(6,720) = 5.366, *p* < 0.001) with Mahalanobis distances that ranged from 0.109 to 0.491. The ‘Syllable’ factor did not incorporate any acoustic measures included in the model from the stepwise regression.

### Study 2 – Listener perception of emotional prosody

**Figure 2:**
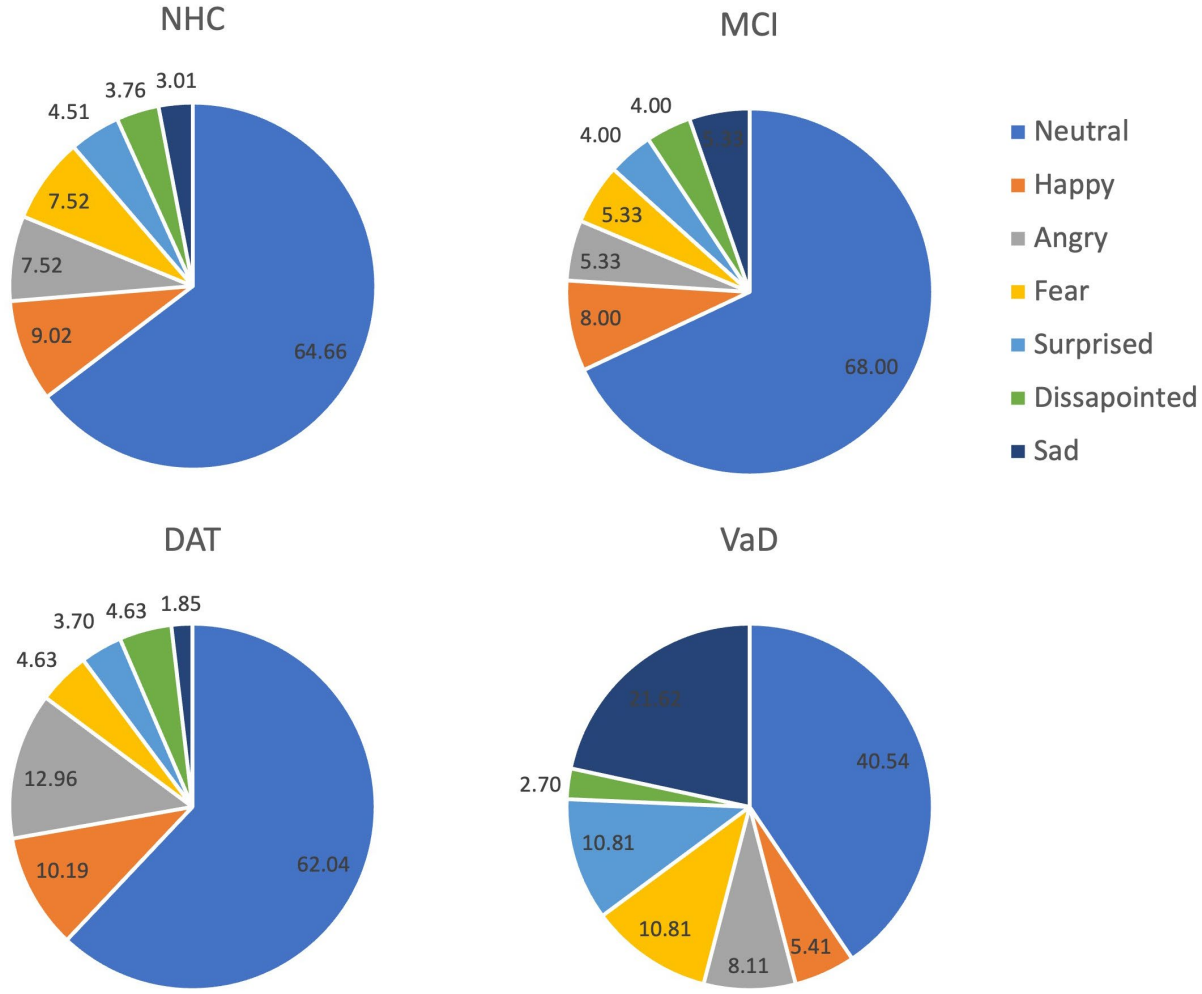
Listener perception (%) of each emotion per group.

Neurotypical listeners perceived neutral prosody in most of the utterances in all speaker groups. The NHC and MCI groups were most similar in terms of the composition of perceived emotions, while the highest number of angry utterances was identified in the DAT group and sad utterances in the VaD group. Table 4 shows the counts of responses and corresponding percentages, and Figure 2 presents the percentage of each perceived emotion.

**Table 4:** Listener perception of emotional prosody.

	NHC	MCI	DAT	VaD
	counts (%)	counts (%)	counts (%)	counts (%)
Neutral	86 (64.66)	51 (68.00)	67 (62.04)	15 (40.54)
Happy	12 (9.02)	6 (8.00)	11 (10.19)	2 (5.41)
Angry	10 (7.52)	4 (5.33)	14 (12.96)	3 (8.11)
Fearful	10 (7.52)	4 (5.33)	5 (4.63)	4 (10.81)
Surprised	6 (4.51)	3 (4.00)	4 (3.70)	4 (10.81)
Disappointed	5 (3.76)	3 (4.00)	5 (4.6)	1 (2.70)
Sad	4 (3.01)	4 (5.33)	2 (1.85)	8 (21.62)

**Figure 3:**
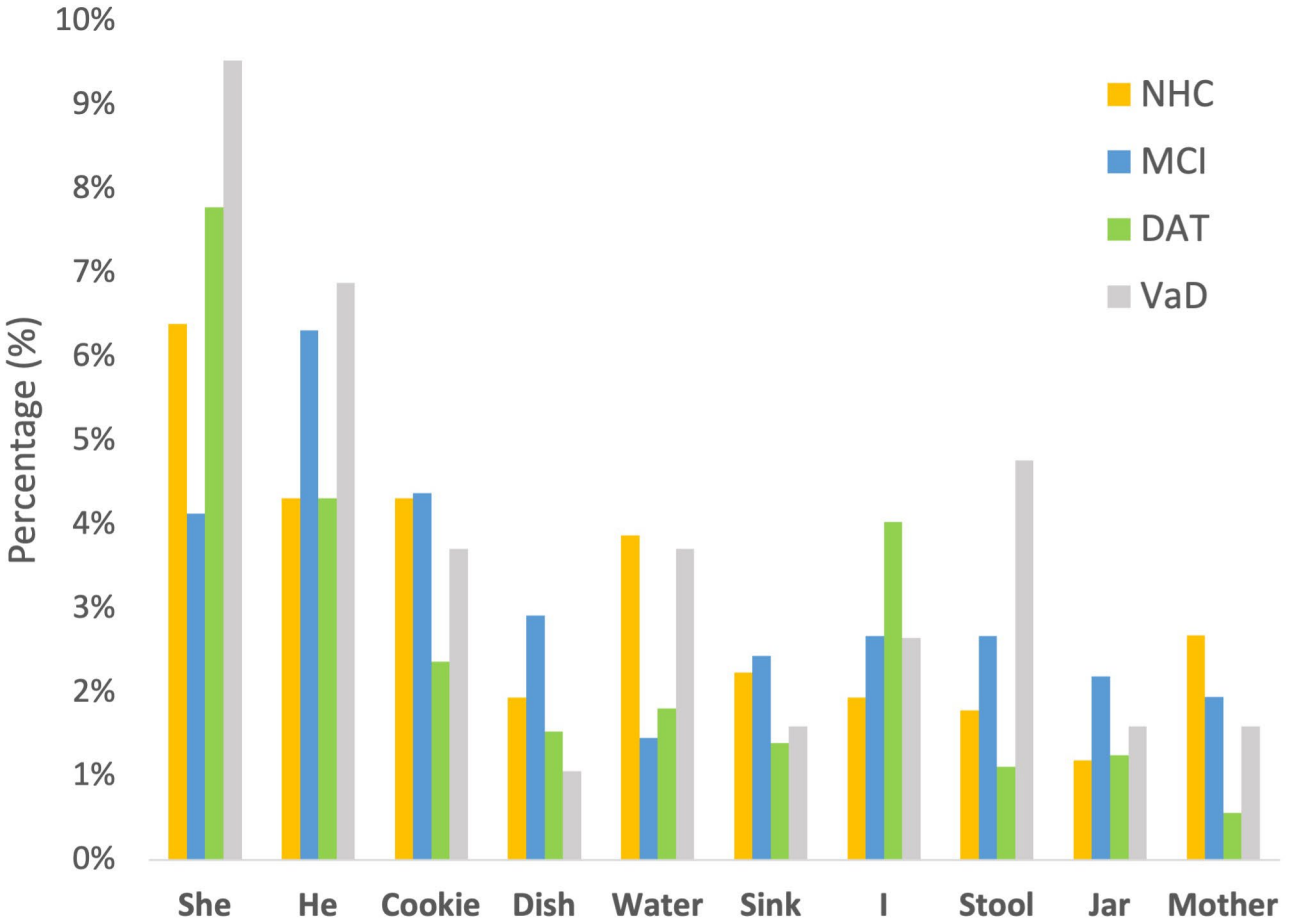
Ten common words used by the four groups of speakers.

To evaluate potential linguistic cues on listener perception of emotional prosody, the words used in the *Cookie Theft* picture description tasks were collected. Words without semantic valence such as *be* verbs and articles were excluded from the collection. As illustrated in Figure 3, the speakers across the 4 groups used similar words to describe the picture. In particular, the 10 most frequently used words constituted approximately 30.67% of NHC speech, 31.07% of MCI speech, 26.11% of DAT speech, and 37.04% of VaD speech, as described in Table 5. Given this finding, the impact of word choice on listener perception of emotion was deemed minimal.

**Table 5:** Words frequently used in the Cookie Theft picture description task.

	Group (%)			
Words	NHC	MCI	DAT	VaD
She	43 (6.39)	17 (4.13)	56 (7.78)	18 (9.52)
He	29 (4.31)	26 (6.31)	31 (4.31)	13 (6.88)
Cookie	29 (4.31)	18 (4.37)	17 (2.36)	7 (3.70)
Dish	13 (1.93)	12 (2.91)	11 (1.53)	2 (1.06)
Water	26 (3.83)	6 (1.46)	13 (1.81)	7 (3.70)
Sink	15 (2.23)	10 (2.43)	10 (1.39)	3 (1.59)
I	13 (1.93)	11 (2.67)	29 (4.03)	5 (2.65)
Stool	12 (1.78)	11 (2.67)	8 (1.11)	9 (4.76)
Jar	8 (1.19)	9 (2.18)	9 (1.25)	3 (1.59)
Mother	18 (2.67)	8 (1.94)	4 (0.56)	3 (1.59)

The logistic regression revealed that Factor 3 of the PCA containing pitch measures as loadings was a significant predictor of emotional prosody. The odds of identifying emotional prosody increased by 22.6% (95% CI: [1.063, 1.418]) for using pitch measures compared to using other measures (i.e., mixed, loudness, rate, and syllable measures). Table 6 presents the outputs of the logistic regression in detail.

**Table 6:** Univariate logistic regression to differentiate emotional prosody from neutral prosody.

Variable	B	SE^a^	Z value	*p*	Exp (B)	95% CI lower	95% CI upper
Intercept	−0.505	0.112	−4.505	<0.001	0.603	0.483	0.750
Mixed	0.083	0.047	1.759	0.078	1.087	0.991	1.194
Loudness	0.024	0.054	0.447	0.655	1.025	0.922	1.141
Pitch	0.204	0.073	2.781	<0.01	1.226	1.063	1.418
Rate	0.057	0.076	0.749	0.454	1.059	0.911	1.231
Syllable	−0.109	0.086	−1.265	0.206	0.896	0.754	1.059

a Standard error.

## Discussion

An accurate diagnosis of cognitive impairment is critical to understand the person’s condition, to establish care and treatment plans and to prepare for expected changes in different areas of daily living. However, the invasive nature and/or high cost of current diagnostic tools make it challenging for people experiencing cognitive impairment to get a precise diagnosis in a timely manner (López-de-Ipiña et al., 2015). Differential diagnosis is particularly important as it guides healthcare professionals and family caregivers in looking into key features and pathology of each type of dementia, so individuals living with the condition can receive the most appropriate treatments and support services that will in turn lead to the highest possible quality of life (Alzheimer’s Association, n.d.). The current research was designed to address this issue by proposing a novel non-invasive and cost-efficient tool for differentiating cognitive impairment phenotypes. To achieve this goal, speech samples of people with different types of cognitive impairment (i.e., MCI, DAT, VaD) and healthy controls were analyzed acoustically for prosodic feature production (Study 1) and neurotypical listeners evaluated emotions conveyed by each utterance (Study 2).

The results of Study 1 demonstrated that acoustic features measured in this study can separate the cognitive impairment types. These features have been associated with emotional prosody (Patel et al., 2011; Pell et al., 2015). Five factors to separate cognitive impairment types were identified using the PCA and 4 of these factors were found salient for differentiating among cognitive impairment groups. Measures included in the 4 factors were the extent of dB changes, the fundamental frequency at the end of utterances, the number of extra syllables in the utterances, and the articulation rate. However, these factors and salient features provided a minimal separation among the cognitive impairment types. In Study 2, the neurotypical listeners perceived distinctive patterns in the utterances of the 4 groups. Although statistical differences were not calculated due to the imbalance of the number of utterances collected across the groups, NHC and MCI showed the most similar patterns. While listeners perceived a neutral prosodic pattern in the majority (>60%) of the utterances in NHC, MCI, and DAT, they indicated that approximately 40% of the utterances of the VaD group were neutral. Across NHC, MCI, and DAT groups, sad prosody consisted of 1 to 3% of all utterances. However, sad emotion was identified in approximately 22% of the VaD utterances. In addition, the listeners perceived that the VaD speakers expressed more utterances in fearful and surprised emotions compared to the other groups. These differences are noteworthy, despite the small number of VaD utterances.

Compiling the results of the two studies, frequency measures were found most critical for the listeners to perceive emotional prosody. This finding agrees with the results of some previous acoustic studies: Bulut and Narayanan (2008) found that the synthetic f0 modification to mean, range, and shape parameters affected the listener’s perception of emotion embedded in the same utterance and Patel et al. (2018) demonstrated that voicing frequency affects the vocal expression of emotion. Although pitch was the strongest perceptual feature, amplitude and timing features also differentiated the four groups in the acoustic analyses. The manipulation of emotional prosody helps the speaker deliver the intention using non-linguistic clues and the listener interpret the intention accurately. This activity requires a wide range of interhemispheric cerebral networks, which is often impaired in people with cognitive impairment (Lian et al., 2018; Qi et al., 2019). The specific domains and severity of the impairment differ across the cognitive impairment groups and therefore, the analysis of emotional prosody can provide a low-cost and non-invasive tool to diagnose different types of cognitive impairment. Despite the strong potential of the analysis of emotional prosody, this line of study has been sparse and shown inconsistent findings. For example, some studies demonstrated that people with dementia struggle when attempting to express emotion (Horley et al., 2010; Haider et al., 2020) and the expression is completed in different ways than neurotypical speakers do (e.g., Meilán et al., 2014; Nevler et al., 2017). Themistocleous et al. (2020) also found that aspects of voice quality and speech fluency of people with MCI and healthy controls differ significantly. Yang et al. (2021) showed correlations between speech features and brain atrophy among people with MCI and DAT and concluded that speech analysis may assist in MCI detection. Other researchers investigated prosody production impairments in people with dementia and reported the potential of acoustic analysis of prosodic features as a dementia diagnostic tool (Kato et al., 2015, 2018; Martinc et al., 2021). However, other studies showed no differences in speech prosody between people with cognitive impairment and those who are healthy (e.g., Testa et al., 2001; Dara et al., 2013 – for spontaneous speech task only, Wright et al., 2018). Nevertheless, these studies either did not report the specific acoustic measures used or assessed a small set of acoustic measures. In addition, they did not clearly distinguish emotional prosody from linguistic prosody.

The present research provides several novel findings: First, it is the first to utilize a large set of acoustic measures that are specifically important for emotion expression to differentially diagnose 3 types of cognitive impairment. In particular, the current research is the first to include the VaD group. According to a recent systematic review (Oh et al., 2021), prosody and dementia studies included DAT and frontotemporal dementia groups only. Second, in this study, emotional prosody was clearly distinguished from linguistic prosody, supported by the neurotypical listeners’ emotion evaluation. It is noteworthy that utterances of the NHC and MCI groups were perceived in a similar pattern while those of the VaD group were unique.

The current research has some limitations: First, the *Cookie Theft* picture description task may not be ideal to elicit emotional responses. Most of the utterances were perceived as neutral by the neurotypical listeners. Unlike the findings of previous studies showing the effectiveness of the *Cookie Theft* picture description task in evoking emotional responses (e.g., Villain et al., 2016; Nevler et al., 2017; Patel et al., 2018; Wright et al., 2018; Haider et al., 2020), the neurotypical listeners involved in this study as emotion raters identified neutral prosody in most of the speakers’ utterances. This leads to the need to develop and validate a more appropriate procedure and/or stimuli. Second, a larger dataset including similar amount of data for each cognitive impairment and healthy group is warranted. Particularly, the listeners’ perception was not statistically tested due to the different number of utterances collected for each group. Despite all the limitations, the findings of the research provide novel and functional implications that are clinically relevant. The findings demonstrate that the analysis of emotional prosody is a promising tool for differential diagnosis of cognitive impairment.

## Data availability statement

The raw data supporting the conclusions of this article is available for verified members of DementiaBank (https://dementia.talkbank.org). Researchers and clinicians working with dementia who are interested in joining the consortium should read the Ground Rules and then send email to macw@cmu.edu with contact information and affiliation. Please include a brief general statement about how you envision using the data.

## Ethics statement

The studies involving human participants were reviewed and approved by Ohio University Institutional Review Board. The patients/participants provided their written informed consent to participate in this study.

## Author contributions

CO: conceptualization (lead), data curation (lead), formal analysis (supporting), funding acquisition (lead), investigation (lead), methodology (lead), project administration (lead), resources (lead), supervision (lead), visualization (lead), writing – original draft (lead), writing – review and editing (supporting). RM: conceptualization (supporting), data curation (supporting), formal analysis (equal), funding acquisition (supporting), investigation (supporting), methodology (supporting), writing – original draft (supporting), writing – review and editing (lead). XW: formal analysis (lead), funding acquisition (supporting), investigation (supporting), methodology (supporting), software (lead), writing – original draft (supporting), writing – review and editing (supporting). MR: investigation (supporting). All authors contributed to the article and approved the submitted version.

## Funding

This research was supported by the Ohio University Research Committee grant.

## Conflict of interest

The authors declare that the research was conducted in the absence of any commercial or financial relationships that could be construed as a potential conflict of interest.

## Publisher’s note

All claims expressed in this article are solely those of the authors and do not necessarily represent those of their affiliated organizations, or those of the publisher, the editors and the reviewers. Any product that may be evaluated in this article, or claim that may be made by its manufacturer, is not guaranteed or endorsed by the publisher.

## Footnotes

^1^https://dementia.talkbank.org

^2^https://app.gorilla.sc/admin/home
